# Supplementary material for: Eco-evolutionary dynamics of gut phageome in wild gibbons (Hoolock tianxing) with seasonal diet variations
Source: Nat Commun. 2024 Feb 10;15:1254. doi: 10.1038/s41467-024-45663-8 (PMC10858875; doi:10.1038/s41467-024-45663-8)
Supplement: Supplementary file 1 — Supplementary Information [file 41467_2024_45663_MOESM1_ESM.pdf]

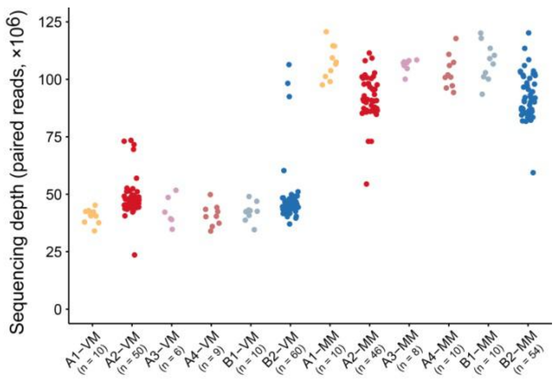

Supplementary Fig. 1 | Sequencing depth (the number of quality-trimmed paired reads in each library) distribution across approaches and individuals. Source data are provided as a Source Data file.

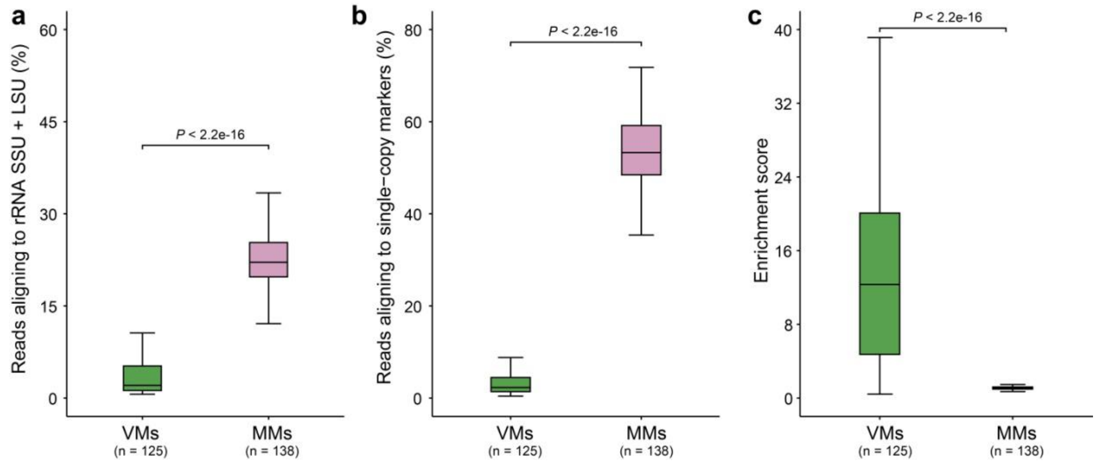

Supplementary Fig. 2 | Comparision of viral metagenomes (VMs) and total metagenomes (MMs) in different samples. a and **b** The percentage of reads of VMs and MMs in different samples aligned to rRNA small and large subunits reference sequences (**a**), and to single-copy bacterial marker genes (**b**). **c** The enrichment score of VMs and MMs derived from ViromeQC software. Data for the three replicates of the ten samples are averaged. Statistical significance is based on non-parametric Wilcoxon t-test (unpaired and two-sided), and the n number (i.e. the sample size used to derive statistics) are provided for each group. Source data are provided as a Source

Data file.

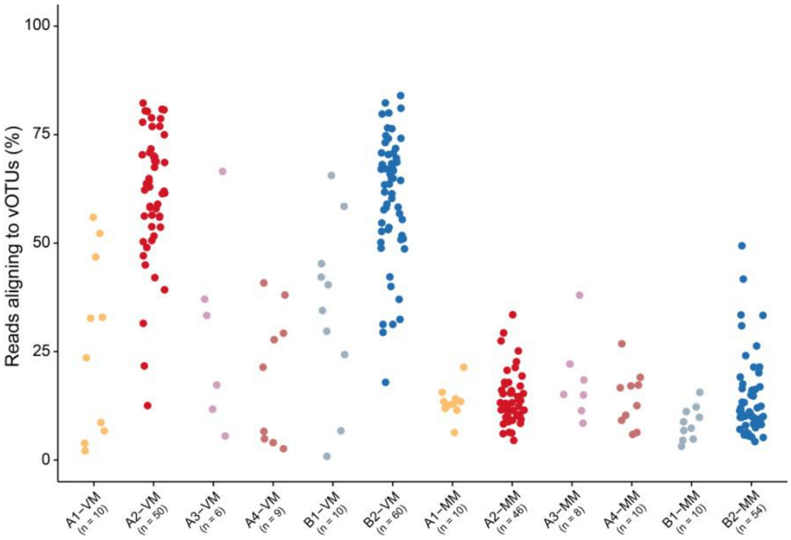

Supplementary Fig. 3 | Percentage of metagenomic reads mapped to the phage genomes. Dot plots show the percentage of reads from VMs and MMs mapped to the representative genomes of VM-vOTUs and MM-vOTUs in each sample, respectively. Source data are provided as a Source Data file.

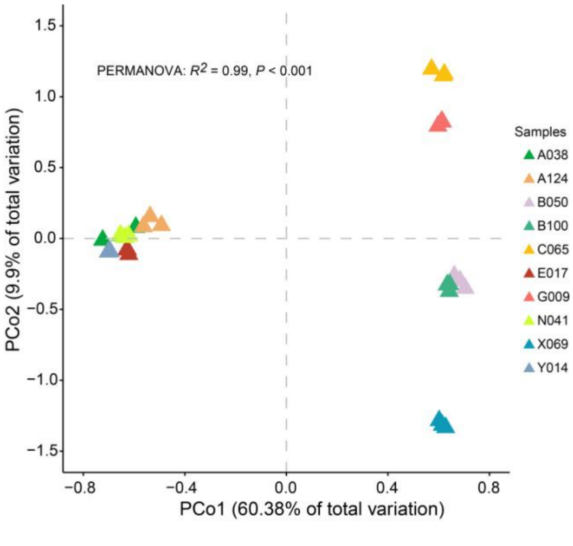

Supplementary Fig. 4 | PCoA of a Bray-Curtis dissimilarity matrix calculated

from the 30 MDA replicates for 10 VM samples. The three independent MDA replicates were closely clustered for each of the ten selected samples in a principal coordinates analysis (PCoA) of community structure of VM-vOTUs, indicating a minor or similar random amplification bias across samples. The permutational multivariate analysis of variance (PERMANOVA) statistics considers samples grouped by samples. Source data are provided as a Source Data file.

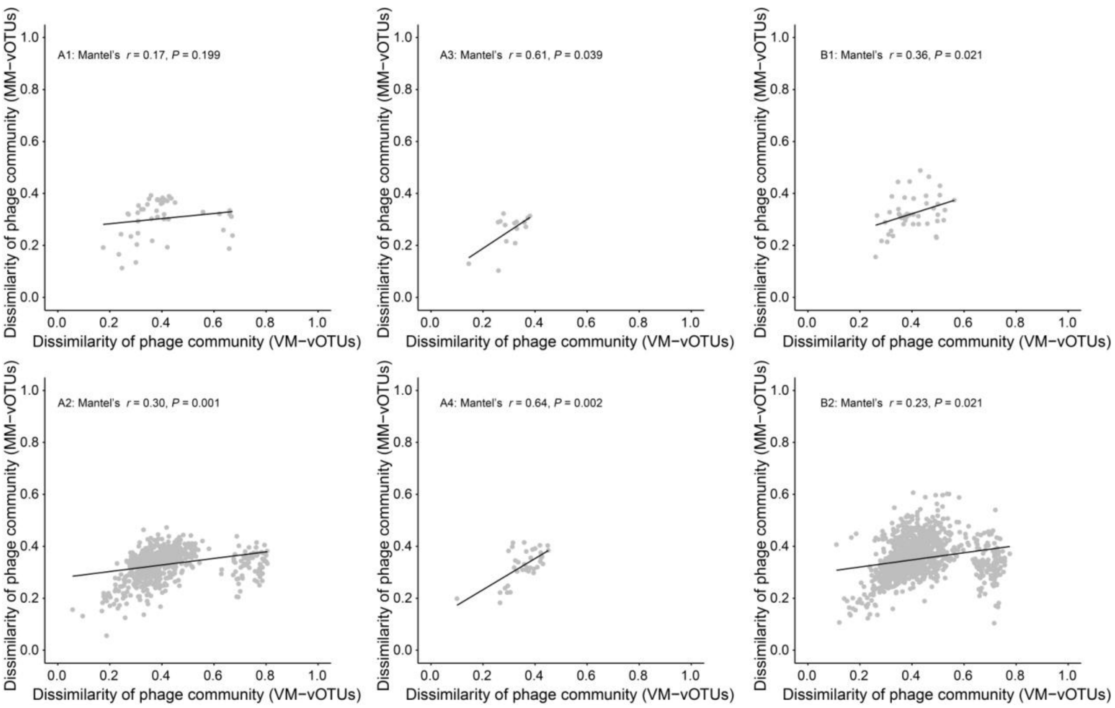

Supplementary Fig. 5 | Mantel's correlation coefficients are used to represent the similarity between Bray-Curtis dissimilarity of community compositions evaluated by VM-vOTUs and MM-vOTUs of the six gibbon individuals. Source data are provided as a Source Data file.

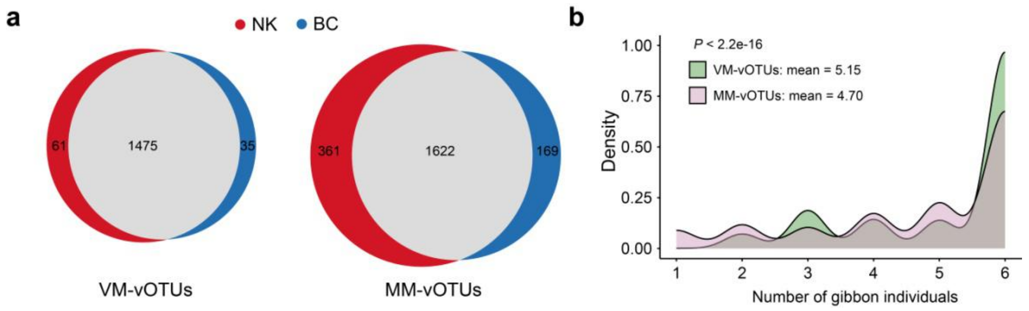

Supplementary Fig. 6 | Distribution of the phage populations across gibbon family groups and individuals. a Venn diagram shows the number of VM-vOTUs (left panel) and MM-vOTUs (right panel) in the two gibbon family groups. B Density plot shows the prevalence (number of gibbon individuals) of VM-vOTUs and MM-vOTUs. Statistical significance is based on non-parametric Wilcoxon t-test (unpaired and two-sided). Source data are provided as a Source Data file.

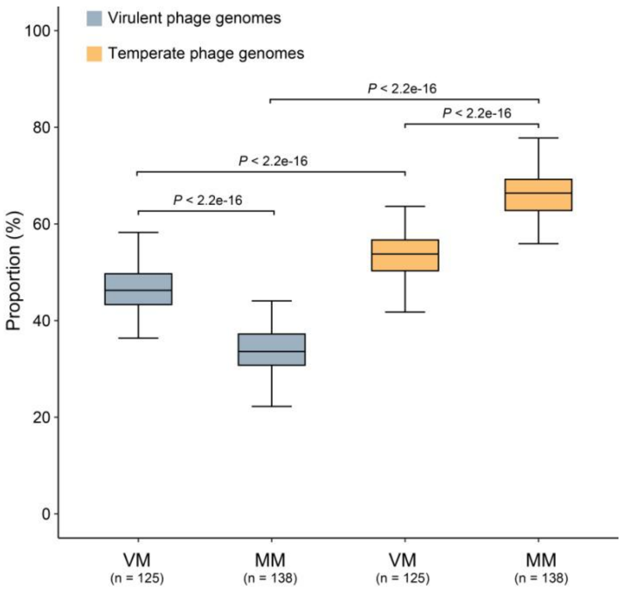

Supplementary Fig. 7 | Proportion of the virulent or temperate phage genomes recovered from VMs and MMs in each sample. Statistical significance is based on non-parametric Wilcoxon t-test (unpaired and two-sided). Source data are provided as

a Source Data file.

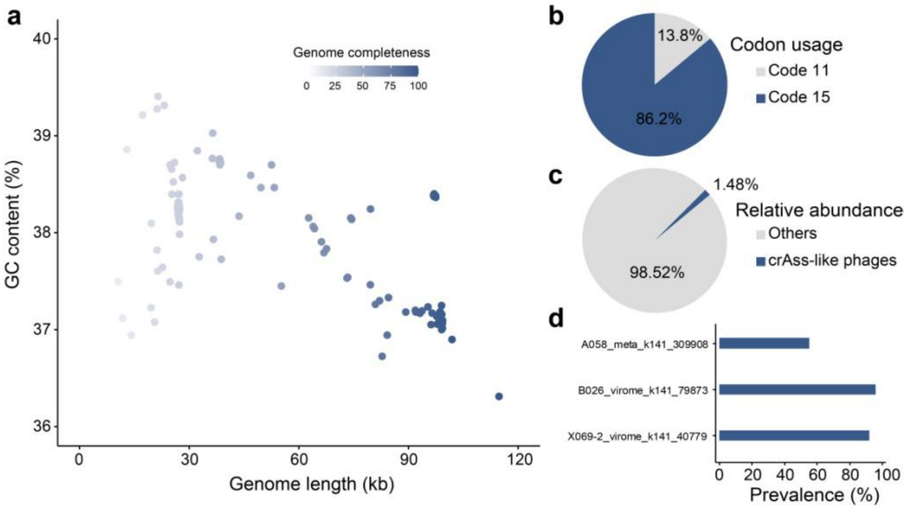

Supplementary Fig. 8 | Genomic features and distribution of the identified crAss-like phages in our study. a Genome size and GC content of the 195 identified crAss-like phages in our study with the dots colored by estimated genome completeness. **b** Predicted codon usage of the 195 phage genomes. **c** and **d** The total relative abundance (**c**) and prevalence (**d**) of the representative genomes of three crAss-like phage populations. Source data are provided as a Source Data file.

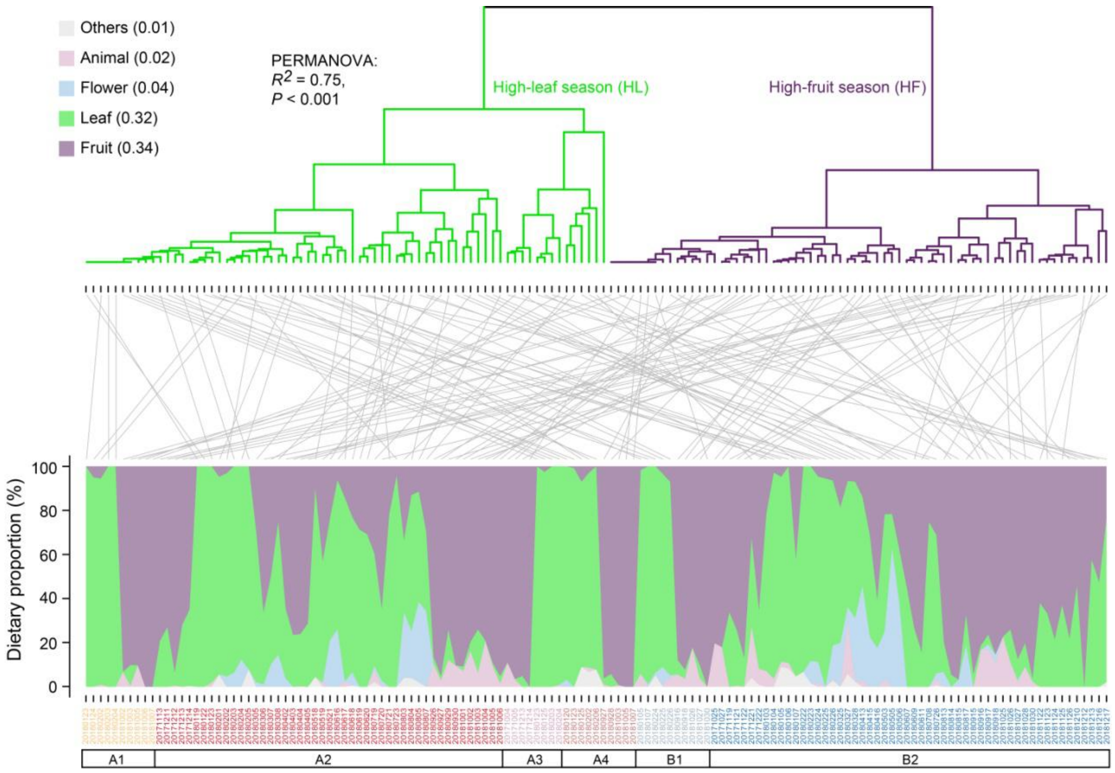

Supplementary Fig. 9 | Seasonal variations in gibbons dietary composition. Hierarchical cluster analysis of dietary compositions for the six gibbons. Numbers in the brackets after the food types indicate the contribution of each food item to the overall dissimilarity between dietary seasons. Source data are provided as a Source Data file.

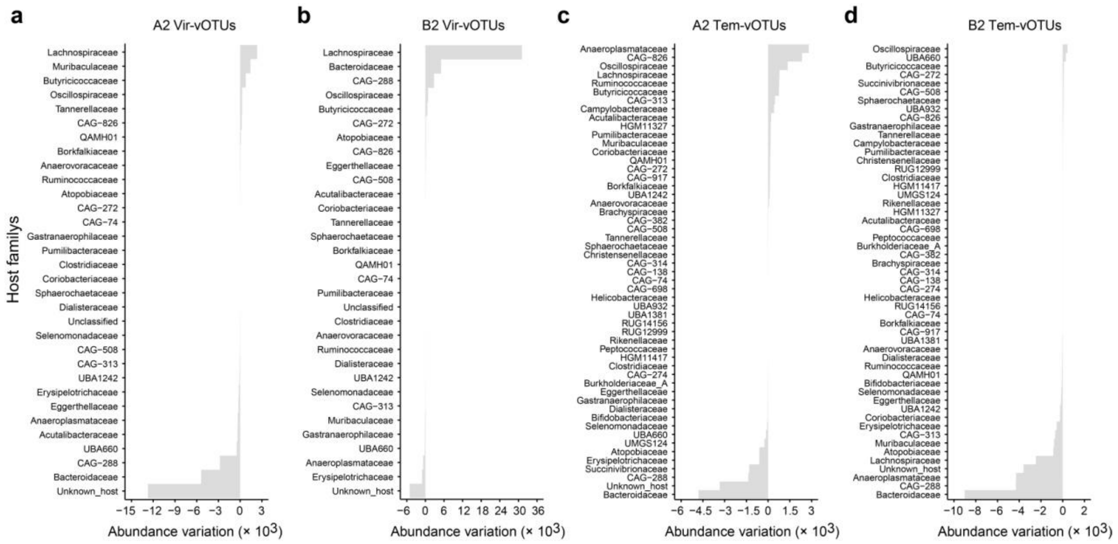

Supplementary Fig. 10 | Phage abundance variation from HF to HL seasons. Bar plots show the variations in the mean abundance of virulent (**a** and **b**) and temperate (**c** and **d**) phage populations grouped by predicted host families from HF to HL seasons. The variation value was calculated by subtracting the mean abundance of phage populations assigned to different host families in HL season from that in HL season. Source data are provided as a Source Data file.

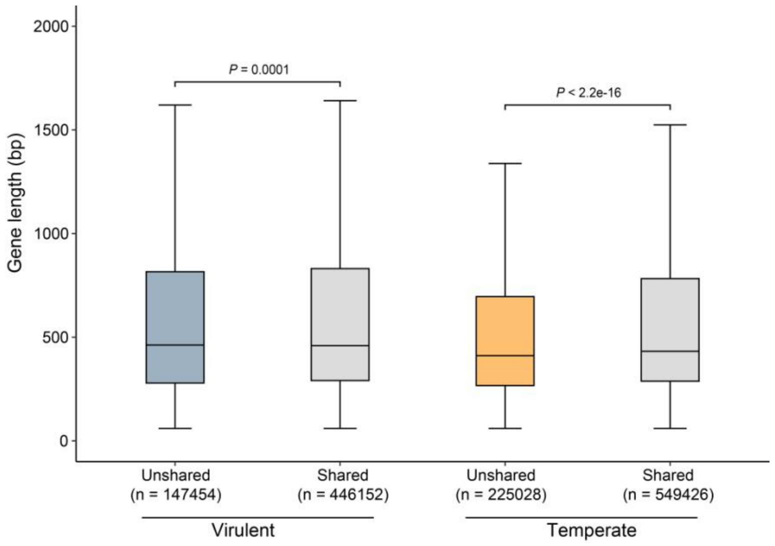

Supplementary Fig. 11 | Boxplot shows the size of genes in shared PCs and unshared PCs on virulent and temperate genomes. Statistical significance is based on

non-parametric Wilcoxon t-test (unpaired and two-sided), and the n number (i.e. the sample size used to derive statistics) are provided for each group. Source data are provided as a Source Data file.

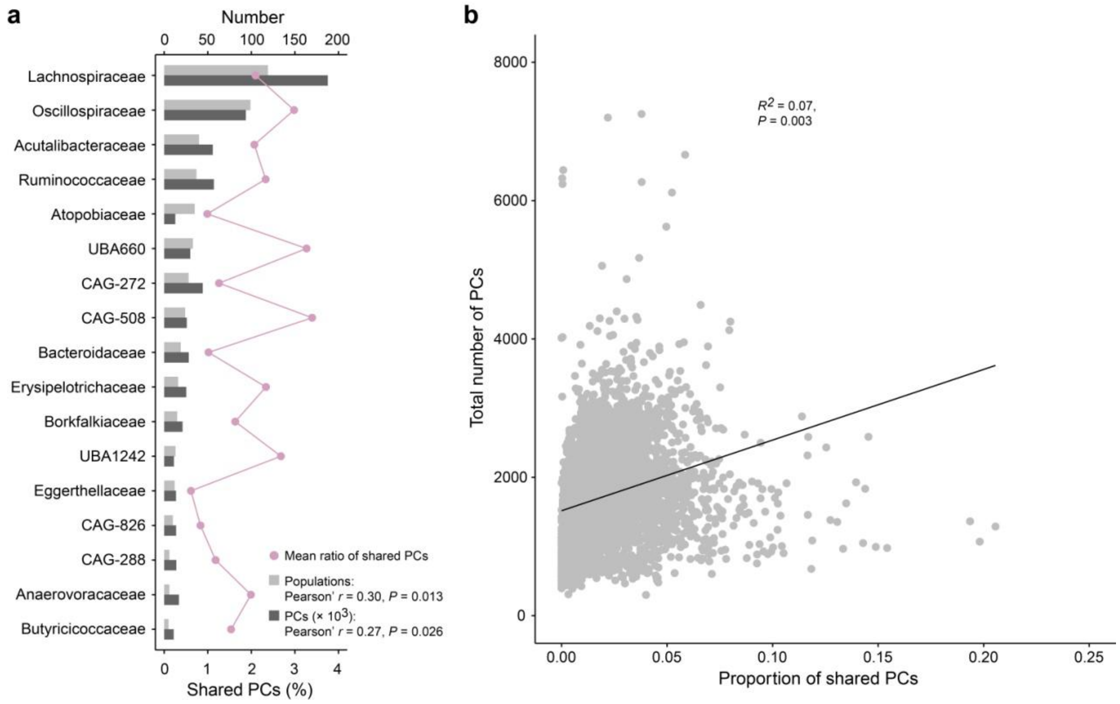

Supplementary Fig. 12 | The proportion of shared PCs in prokaryotic families and genomes. a Barplots show the number of populations and PCs in each host family, while the line chart indicate the mean proportion of shared PCs for each prokaryotic family. Pearson's correlations between the mean proportion of shared PCs and the number of populations or PCs are presented. **b** Correlations between the proportion of shared PCs and the total number of PCs across the 10,567 prokaryotic genomes. The adjusted R^2 values and best-fit lines for the linear regressions are presented. Source data are provided as a Source Data file.

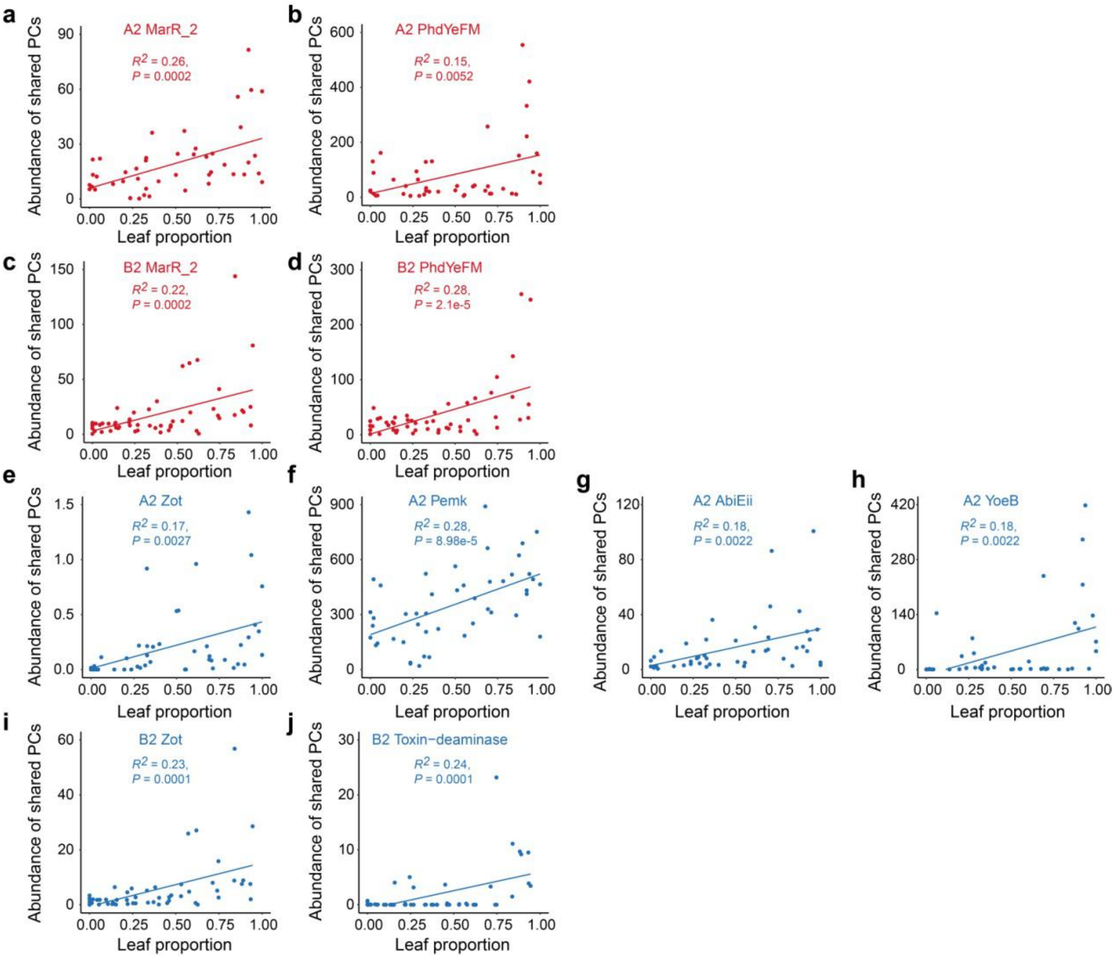

Supplementary Fig. 13 | Correlations between the abundance of shared PCs contained the diet-responsive toxin/antitoxin genes and leaf proportion. Correlations between leaf proportion and the abundance of antitoxin genes in shared PCs in gibbons A2 (**a** and **b**) and B2 (**c** and **d**). Correlations between leaf proportion and the abundance of toxin genes in shared PCs in gibbons A2 (**e, f, g,** and **h**) and B2 (**i** and **j**). The adjusted R^2 values and best-fit lines for the linear regressions are presented. Source data are provided as a Source Data file.

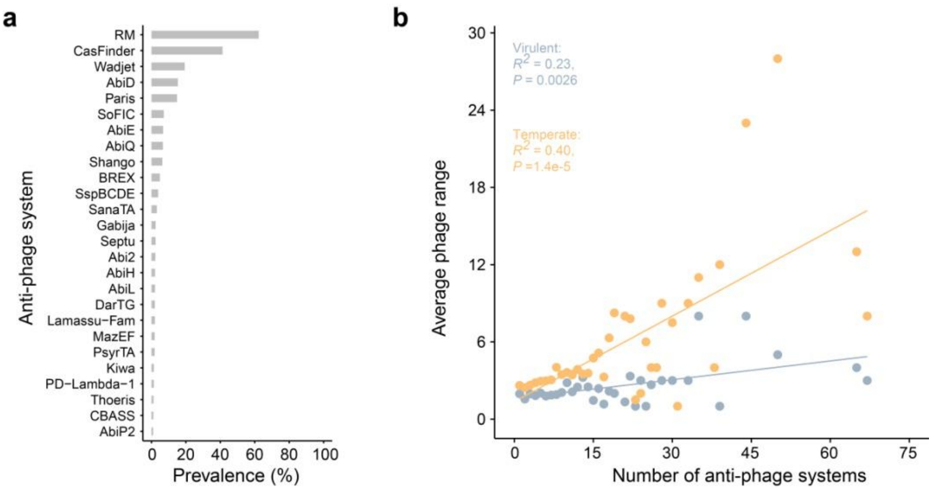

Supplementary Fig. 14 | The distribution of Anti-phage systems and its associations with phage ranges. a Barplot shows the number of prokaryotic genomes (prevalence) that contained diverse anti-phage systems. **b** Correlations between the number of anti-phage systems and the mean virulent and temperate phage ranges. The mean phage ranges were calculated by averaging the number of predicted phage populations for prokaryotic genomes with the same number of anti-phage systems. The adjusted R^2 values and best-fit lines for the linear regressions are presented. Source data are provided as a Source Data file.

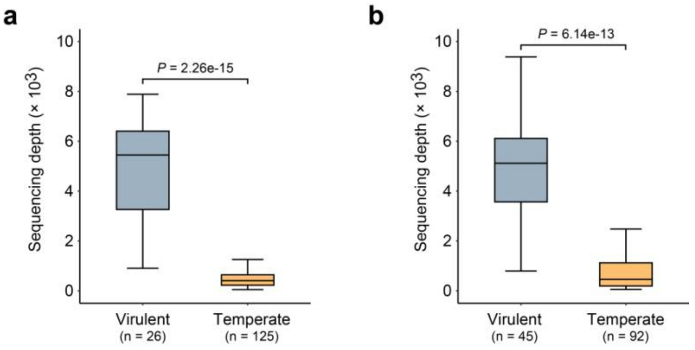

Supplementary Fig. 15 | Comparison of the sequencing depth of virulent and temperate genomes with a minimum sequencing depth of 10-fold across the samples

in gibbons A2 (**a**) and B2 (**b**). Statistical significance is based on non-parametric Wilcoxon t-test (unpaired and two-sided). Source data are provided as a Source Data file.

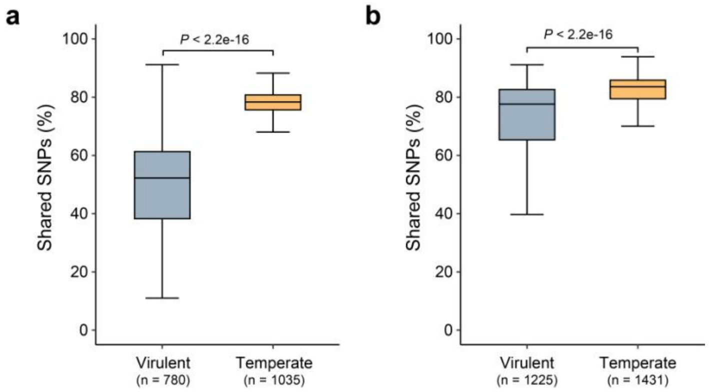

Supplementary Fig. 16 | Comparison of the proportion of shared SNPs identified on virulent and temperate genomes in gibbons A2 (**a**) and B2 (**b**). Statistical significance is based on non-parametric Wilcoxon t-test (unpaired and two-sided). Source data are provided as a Source Data file.

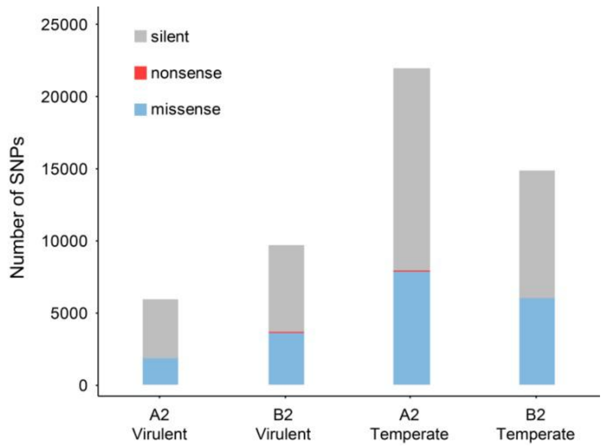

Supplementary Fig. 17 | Number of SNPs colored with different types identified on virulent and temperate phage genomes in gibbons A2 and B2. Source data are provided as a Source Data file.
