# Supplementary material for: Eco-evolutionary dynamics of gut phageome in wild gibbons (Hoolock tianxing) with seasonal diet variations
Source: Nat Commun. 2024 Feb 10;15:1254. doi: 10.1038/s41467-024-45663-8 (PMC10858875; doi:10.1038/s41467-024-45663-8)
Supplement: Supplementary file 3 — Description of Additional Supplementary Files [file 41467_2024_45663_MOESM3_ESM.pdf]

Description of Additional Supplementary Files

Supplementary Data Legends

Title: Supplementary Data 1

Description: Abiotic and biotic data for the 139 fecal samples from the six female gibbons.

Title: Supplementary Data 2

Description: Detailed information of predicted phage genomes identified in the viral metagenomes (VMs) and microbial metagenomes (MMs).

Title: Supplementary Data 3

Description: Normalized abundance of the 1,571 VM-vOTUs in 125 feces sequenced with viral metagenomes.

Title: Supplementary Data 4

Description: Normalized abundance of the 2,153 MM-vOTUs in 138 bulk metagenomic DNA libraries.

Title: Supplementary Data 5

Description: Normalized abundance of the 1,073 Vir-vOTUs in 125 feces sequenced with viral metagenomes.

Title: Supplementary Data 6

Description: Normalized abundance of the 2,157 Tem-vOTUs in 138 bulk metagenomic DNA libraries.

Title: Supplementary Data 7

Description: The non-parametric test for the abundance of each virulent or temperate phage populations between the two dietary seasons in gibbons A2 and B2.

Title: Supplementary Data 8

Description: Virus-host linkages predicted by shared genomic matches with host genomes and protospacer-spacer matches.

Title: Supplementary Data 9

Description: Lists of Vir-PCs and Tem-PCs annotated as genes related to toxin/antitoxin proteins.

Title: Supplementary Data 10

Description: Lists of identified SNPs located on predicted genes of virulent and temperate population genomes in the gibbons A2 and B2.

Title: Supplementary Data 11

Description: Detailed accession number of the viral metagenomes and microbial metagenomes generated in this study
