# Supplementary material for: Eco-evolutionary dynamics of gut phageome in wild gibbons (Hoolock tianxing) with seasonal diet variations
Source: Nat Commun. 2024 Feb 10;15:1254. doi: 10.1038/s41467-024-45663-8 (PMC10858875; doi:10.1038/s41467-024-45663-8)
Supplement: Supplementary file 5 — Reporting Summary [file 41467_2024_45663_MOESM5_ESM.pdf]

nature portfolio

Corresponding author(s): Peng-Fei Fan

Last updated by author(s): Jan 14, 2024

Reporting Summary

Nature Portfolio wishes to improve the reproducibility of the work that we publish. This form provides structure for consistency and transparency in reporting. For further information on Nature Portfolio policies, see our Editorial Policies and the Editorial Policy Checklist.

Statistics

For all statistical analyses, confirm that the following items are present in the figure legend, table legend, main text, or Methods section.

n/a	Confirmed	
☐	☒	The exact sample size (*n*) for each experimental group/condition, given as a discrete number and unit of measurement
☐	☒	A statement on whether measurements were taken from distinct samples or whether the same sample was measured repeatedly
☐	☒	The statistical test(s) used AND whether they are one- or two-sided<br>*Only common tests should be described solely by name; describe more complex techniques in the Methods section.*
☒	☐	A description of all covariates tested
☐	☒	A description of any assumptions or corrections, such as tests of normality and adjustment for multiple comparisons
☐	☒	A full description of the statistical parameters including central tendency (e.g. means) or other basic estimates (e.g. regression coefficient) AND variation (e.g. standard deviation) or associated estimates of uncertainty (e.g. confidence intervals)
☐	☒	For null hypothesis testing, the test statistic (e.g. *F*, *t*, *r*) with confidence intervals, effect sizes, degrees of freedom and *P* value noted<br>*Give P values as exact values whenever suitable.*
☒	☐	For Bayesian analysis, information on the choice of priors and Markov chain Monte Carlo settings
☐	☒	For hierarchical and complex designs, identification of the appropriate level for tests and full reporting of outcomes
☐	☒	Estimates of effect sizes (e.g. Cohen's *d*, Pearson's *r*), indicating how they were calculated

Our web collection on statistics for biologists contains articles on many of the points above.

Software and code

Policy information about availability of computer code

Data collection	crAss-like phages sequences: Guerin et al., 2018. https://doi.org/10.1016/j.chom.2018.10.002 (Data S1);<br>crAss-like phages sequences: Yutin et al., 2021. ftp://ftp.ncbi.nih.gov/pub/yutinn/crassfamily_2020/;<br>Lak phages sequences: Devoto et al., 2019. NCBI: PRJNA491720;<br>Lak phages sequences: Crisci et al., 2021. https://figshare.com/articles/dataset/34_new_Lak_phage_genomes/13493721;<br>Virus Orthologous Group database release 219: http://vogdb.org;<br>Pfam database v36.0: https://ftp.ebi.ac.uk/pub/databases/Pfam/current_release.
Data analysis	Megahit v1.2.9, fastp v0.21.0, MetaBAT v2.12.1, Bowtie2 v2.2.9, ViromeQC v1.0, RefineM v0.0.24, dRep v3.2.2, BamM v1.7.3, VirSorter v2.2.0, CheckV v0.6.0, Prodigal v2.6.3, Prodigal v2.50, Deephage v1.0, metaCRT v1.2, BLASTn 2.5.0, PhaGCN v2.0, cd-hit v4.8.1, CheckM2 v1.0.2, GTDB-Tk v2.3.0, DefenseFinder v1.2.0, hmmsearch v3.3.2, samtools v 1.17, bcftools v1.17, SNPEff v5.2, R v4.0.3, vegan v2.5-5, Hmisc v4.2-0, ggplot2 v3.1.1, stats v4.0.3.

For manuscripts utilizing custom algorithms or software that are central to the research but not yet described in published literature, software must be made available to editors and reviewers. We strongly encourage code deposition in a community repository (e.g. GitHub). See the Nature Portfolio guidelines for submitting code & software for further information.

Data

Policy information about availability of data

All manuscripts must include a data availability statement. This statement should provide the following information, where applicable:

- Accession codes, unique identifiers, or web links for publicly available datasets
- A description of any restrictions on data availability
- For clinical datasets or third party data, please ensure that the statement adheres to our policy

Raw viral metagenomic sequencing data have been deposited in NCBI BioProject database under accession code PRJNA831632 [https://www.ncbi.nlm.nih.gov/bioproject/PRJNA831632]. Microbial metagenomic sequencing data have been deposited in the National Genomics Data Center, China National Center for Bioinformation or Beijing Institute of Genomics, Chinese Academy of Sciences under the accession code PRJCA012504 [https://ngdc.cncb.ac.cn/bioproject/browse/PRJCA012504]. Biosample accession numbers for individual prokaryotic genomes are listed in Supplementary Data 11. The data that support the findings of this study are available from https://doi.org/10.6084/m9.figshare.20099621. Source data are provided with this paper.

Research involving human participants, their data, or biological material

Policy information about studies with human participants or human data. See also policy information about sex, gender (identity/presentation), and sexual orientation and race, ethnicity and racism.

Reporting on sex and gender	N/A
Reporting on race, ethnicity, or other socially relevant groupings	N/A
Population characteristics	N/A
Recruitment	N/A
Ethics oversight	N/A

Note that full information on the approval of the study protocol must also be provided in the manuscript.

Field-specific reporting

Please select the one below that is the best fit for your research. If you are not sure, read the appropriate sections before making your selection.

☐ Life sciences ☐ Behavioural & social sciences ☒ Ecological, evolutionary & environmental sciences

For a reference copy of the document with all sections, see nature.com/documents/nr-reporting-summary-flat.pdf

Ecological, evolutionary & environmental sciences study design

All studies must disclose on these points even when the disclosure is negative.

Study description	In this study, we collected fresh, individual-resolved, and time-series feces (n = 139) from six wild gibbons (Hoolock tianxing) with significant diet variations, and fully recovering the virulent and temperate phage genomes with two complementary metagenomic approaches. Our study show tight couplings in abundance, functions, and evolutionary dynamics between phages and their hosts in the context of gibbon diet variations, providing initial insights into the mechanisms by which phages has helped the wild animals to thrive in changing environments.
Research sample	We chosen two skywalker hoolock gibbon family groups (40 km apart) in Mt. Gaoligong National Nature Reserve, Yunnan, P.R. China: group NK (one adult male, one adult female, one infant, and one juvenile) at Nankang (24°49’N, 98°46’E) , and group BC (one adult male and one adult female) at Banchang (25°12’N, 98°46’E). These two wild gibbon groups were well habituated to observers and all individuals were distinguished by their fur color, body size, and shape of the white eye brow.
Sampling strategy	Fecal samples were stochastically collected during follows. Once a defecation occurred, the landing location was immediately locked and 2-3 mL non-contaminated feces were collected within 5 minutes. Samples were sealed in 50 mL sterile tubes with 95% ethanol and then transported to the laboratory where they were stored at -80°C. Finally, 139 fecal samples with parallel feeding behavior data of the six gibbons were collected. We also recorded all feeding activities (5-min scan and ad libitum), recording the start time, end time, food species and food type (e.g., leaves, fruit, flower, animals, and others) of each feeding bout for the focal individual. To calculate the dietary composition for each gibbon, the recorded feeding times allocated to the same food type were added and then divided by the total feeding time in a full-day.
Data collection	The viral metagenomes and total metagenomes library was prepared by S.M.G. and sequenced on an Illumina NovaSeq 6000 platform (150 bp paired-end reads). Sequencing reads were processed by S.M.G. for quality control, scaffolds assembly, genome binning, viral sequence identification, and ecological analyses.

Timing and spatial scale	To record the dietary data of the wild gibbons for different seasons, all samples were collected during Oct-2017 to Dec-2017 in Mt. Gaoligong National Nature Reserve, Yunnan, P.R. China. We followed the gibbon individuals of each group for average eight consecutive days per month, each day from the time gibbons left their sleeping trees in the morning until they returned to the trees in the afternoon.
Data exclusions	To explore the potential impact of dietary changes on gut viruses, only feces with parallel dietary data collected in the previous day were used in this study. Finally, a total of 139 feces with matched full-day dietary data were obtained and used for subsequent processing.
Reproducibility	This experiment has not been reproduced but can be reproduced with the methods and supplementary data and codes provided in the manuscript.
Randomization	Not applicable as sampling was performed from wild gibbons and not treatments were applied
Blinding	Not applicable as sampling was performed from wild gibbons and not treatments were applied

Did the study involve field work? ☒ Yes ☐ No

Field work, collection and transport

Field conditions	The mean annual precipitation was 1,633 mm and 1,655 mm and mean annual temperature was 13.2° and 13.1° at Banchang and Nankang, respectively. The vegetation at these two sites is characterized by mid-montane evergreen broad-leaved forests between 1,600 - 2,700 m above sea level. (Fei et al. Anim. Cogn. 2022. https://doi.org/10.1007/s10071-022-01600-0)
Location	Location information is available in Fig. 1a. Specifically, the two gibbon groups live in group NK (one adult male, one adult female, one infant, and one juvenile) at Nankang (24°49′N, 98°46′E), and group BC (one adult male and one adult female) at Banchang (25° 12′N, 98°46′E).
Access & import/export	At the time of collection, all fecal samples were obtained with permission from Mt. Gaoligong National Nature Reserve. The import/export statement was not relevant to our samples because they were transported and processed in China.
Disturbance	No disturbances or harms are caused by this study

Reporting for specific materials, systems and methods

We require information from authors about some types of materials, experimental systems and methods used in many studies. Here, indicate whether each material, system or method listed is relevant to your study. If you are not sure if a list item applies to your research, read the appropriate section before selecting a response.

Materials & experimental systems

n/a	Involved in the study	
☒	☐	Antibodies
☒	☐	Eukaryotic cell lines
☒	☐	Palaeontology and archaeology
☐	☒	Animals and other organisms
☒	☐	Clinical data
☒	☐	Dual use research of concern
☒	☐	Plants

Methods

n/a	Involved in the study	
☒	☐	ChIP-seq
☒	☐	Flow cytometry
☒	☐	MRI-based neuroimaging

Animals and other research organisms

Policy information about studies involving animals; ARRIVE guidelines recommended for reporting animal research, and Sex and Gender in Research

Laboratory animals	N/A
Wild animals	We followed six skywalker hoolock gibbons (Hoolock tianxing) in Mt. Gaoligong National Nature Reserve, Yunnan, P.R. China for average eight consecutive days per month from October 2017 to December 2018. During follows, we recorded all feeding activities and collected non-contaminated feces for each gibbon.
Reporting on sex	Gibbon group NK includes one adult male, one adult female, one infant, and one juvenile, and group BC includes one adult male and one adult female.
Field-collected samples	Once a defecation occurred, the landing location was immediately locked and 2-3 mL non-contaminated feces were collected within

Field-collected samples	5 minutes. Samples were sealed in 50 mL sterile tubes with 95% ethanol and then transported to the laboratory where they were stored at -80°C.
Ethics oversight	Our field studies were conducted under the approval of Yunnan Gaoligongshan National Nature Reserve, Baoshan Bureau.

Note that full information on the approval of the study protocol must also be provided in the manuscript.
